# Development and validation of the *thyroidectomy training box*: cervical simulator for training endoscopic transoral thyroidectomy

**DOI:** 10.1590/acb397624

**Published:** 2024-11-08

**Authors:** Valdenor Neves Feitosa, Taís Vasconcelos Cidrão, Ingrid Arruda Castro, Klayton Coelho de Souza, Sarah Rodrigues Chaves Martins, Francisco Everton Pereira da Silva, Luiz Gonzaga Moura

**Affiliations:** 1 Centro Universitário Christus – Fortaleza (CE) – Brazil.

**Keywords:** General Surgery, Thyroid Gland, Mentoring, Malingering

## Abstract

**Purpose:**

To develop and validate a trans oral endoscopic thyroidectomy vestibular approach (TOETVA) simulator.

**Methods:**

The first phase of the project consisted of designing and developing a transoral thyroid surgery simulator based on real surgeries. The product has the oral cavity for attaching the three trocars and the cervical part containing the thyroid and adjacent structures. In the second phase, the simulator was validated by specialists who performed an endoscopic thyroidectomy procedure. They all filled a questionnaire about the simulator and the simulation based on the Likert scale.

**Results:**

The simulator consists of a console similar to a human bust and a high-resolution camera system connected to a 22-inch monitor. The simulator had excellent results in the visual evaluation (*face validity*), with 100% of responses between good and excellent for the following characteristics: synthetic structures, design, visibility of the surgery field, resistance, resilience, *fulcrum* effect, ergonomics, surgical material, and practicality. The last three were rated higher, with more votes for excellent. For *content validity*, the items that received the best ratings were, precisely, the steps relating to the surgical procedure: opening the intermuscular midline, isthmotomy, and thyroidectomy.

**Conclusions:**

The thyroidectomy training box showed great ability to simulate a TOETVA, with satisfactory evaluations concerning its visual and content validation.

## Introduction

Modern thyroidectomy began in the 19th century with Theodor Billroth (1829-1894) and Theodor Kocher (1841-1917), the latter being considered the father of thyroid surgery for the technique developed by him^1^. His technique has been used since, giving its name to the incision made (Kocher’s incision).

Over time, the appearance of the cervical scar has become one of the main postoperative complaints^2^. Its unsatisfactory appearance can significantly affect the patient’s quality of life and social interaction^3^.

Thus, some techniques have been created to reduce or hide the scars, such as minimally invasive video-assisted thyroidectomy (MIVAT), by Miccoli et al. in 1999^4^. Although the scar is reduced, it is still visible, and there is the possibility of undesirable scarring^5^.

Faced with this problem, trans oral endoscopic thyroidectomy vestibular approach (TOETVA) emerged as a viable option^6^^,^ 7, gaining notoriety with Anuwong et al.’s results^8^^,^ 9. After its popularization, the technique has been attracting new practitioners who need to solve some problems to obtain excellent results, such as the availability of endoscopic material and overcoming the learning curve^10^. Anuwong et al. suggested that the minimum number of patients to qualify for the technique should be 7–10 cases^8^.

Knowing that there is no training model or simulator for TOETVA in the literature until the publication of this study, and aware of the great benefits of surgical training, such as gaining surgical skills and reducing operative time^1^^,^ 11, this study aimed to create and validate a simulator for transoral thyroid surgery.

## Methods

The creation of the simulator, up to its final stage, consisted of two phases: design, development, and improvement; and validation with experts.

### Phase 1: development

**Figure 1:**
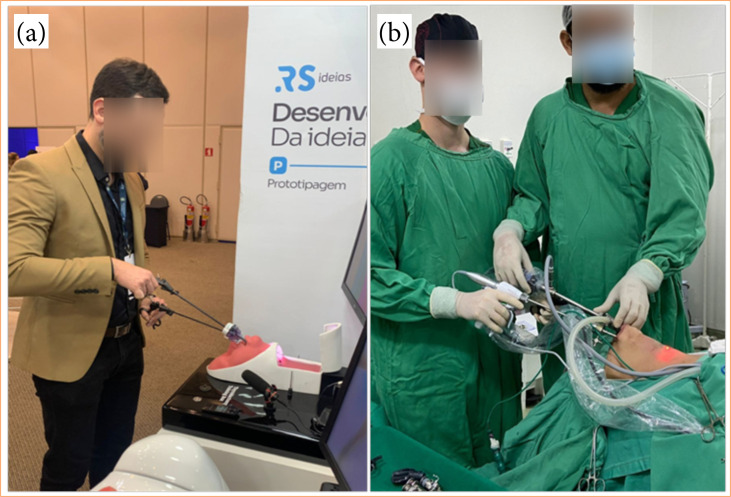
Similarity between the prototype and surgery on a real patient.

Source: Elaborated by the authors.

Created in partnership with RS Soluções Médicas, the simulator was initially developed after observing several TOETVA surgeries. The external structure was based on a human being in the horizontal dorsal decubitus position with cervical hyperextension and a view of the surgeon from the head towards the feet (Fig. 1).

For the internal framework, it was initially planned to place important surgical steps, such as opening the midline to access the thyroid store and identifying and dissecting the thyroid, recurrent laryngeal nerves, and parathyroid glands.

**Figure 2:**
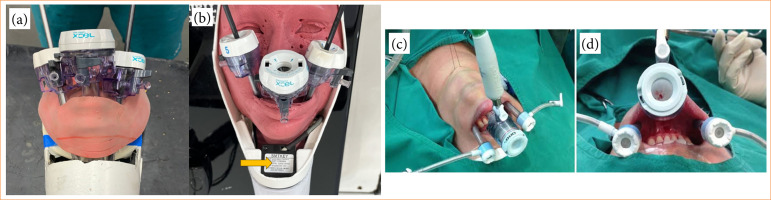
Finished simulator. (a and b) Comparison of trocar distribution, **(b)** showing more space between them in the new mold. Yellow arrow showing new camera position. (c and d) Real surgery demonstration of trocar distribution, **(b)** more similar to the larger mold.

Source: Elaborated by the authors.

Once the external and internal parts of the simulator had been defined, we moved on to printing the mannequin containing the neck and head. At this point, clamp positioning, triangulation, depth, and ergonomics were improved. As in human surgery, the trocars are inserted transorally, so the mouth also needed to be more malleable to simulate real freedom of movement, which is why the head was made from the same material as the internal soft tissues. The material used to mimic the head, pre-thyroid muscles, thyroid, and trachea was a thermoplastic elastomer (TPE) polymer (Fig. 2).

One of the difficulties was finding the ideal position for the camera, since in real surgery there is always an assistant to hold the videoendoscopy optics. As a simulator is being developed to train residents and surgeons, it is known that there will not always be an assistant at the time of the simulation, so it was decided to leave a fixed camera on the topography of the chin fixed to support. If the surgeon wants to train with the assistant, s/he inserts an optic through the central trocar.

The creation of a *workstation* was also conceived to add all the technological support to help with simulation training, with the coupling of a video set that can record images and audio, as well as being used to transmit in real time to other devices connected to the internet.

### Phase 2: validation

For the validation stage, 10 surgeons with proven experience in TOETVA were randomly recruited, with more than 10 surgeries as the suggested cut-off^8^, and were subjected to visual and functional evaluation questionnaires about the simulator and the simulation. It is important to notice that the participants were invited spontaneously and randomly during the exhibition of the simulator at the Brazilian Congress of Head and Neck Surgery held in September 2023 in João Pessoa, Paraíba, Brazil.

This group may represent approximately 5% of the experts who have already carried out at least one TOETVA in Brazil up to the date of the test^12^. The estimate of experts to evaluate the simulator was based on references that suggest a minimum of six evaluators^13^, with no significant additional benefit when this number exceeds twelve people^14^.

All participants signed an informed consent form. The study was approved by the Unichristus Research Ethics Committee (protocol CEP 69631523.6.0000.5049) under Resolution no. 466/12 of the National Health Council.

The inclusion criteria were: residency training recognized by the Ministry of Education in Head and Neck Surgery and experience in more than 10 TOETVA thyroid surgeries^8^. Exclusion criteria was refusal to take part in the study or having performed less than 10 TOETVAs.

The simulations were recorded and timed, starting when the first trocar was inserted. The end of the procedure consisted of total removal of the thyroid via the transoral route.

After the simulation, the surgeons filled in a structured form online in 24 hours, without the author being present, in which data was collected in order to measure and provide knowledge about the participant’s training and level of graduation, as well as their surgical skills and their experience with video surgeries. They then answered questions about the simulator and the simulation, ending with suggestions and criticisms.

### Simulator evaluation

In order to evaluate the simulator, questions were asked about its characteristics using a Likert scale, in which “poor” corresponded to the worst evaluation, and “excellent” to the best evaluation (Table 1).

**Table 1:** Evaluation of the simulator and simulation characteristics.

Characteristics	Poor	Bad	Regular	Good	Excellent
Ability to simulate a trans oral endoscopic thyroidectomy vestibular approach	1	2	3	4	5
Synthetic structures	1	2	3	4	5
Tweezers and surgical equipment	1	2	3	4	5
Video equipment	1	2	3	4	5
Visual appearance	1	2	3	4	5
Simulator design	1	2	3	4	5
Adequate depth	1	2	3	4	5
Ergonomics and positioning	1	2	3	4	5
Visibility of the operating field	1	2	3	4	5
Distribution of portals	1	2	3	4	5
Material strength	1	2	3	4	5
Material resilience (ability to deform and reform)	1	2	3	4	5
Fulcrum effect	1	2	3	4	5
Simulator practicality	1	2	3	4	5

Source: Elaborated the authors.

### Simulation evaluation

A questionnaire was used to evaluate the simulation, which scored each stage of the surgery based on a Likert scale (Table 2). This table only includes the steps performed during the procedure. Each procedure was recorded on video and timed for comparison purposes and to establish a standard that could be used to train new surgeons and validate the simulator in future tests.

**Table 2:** Evaluation of the simulation steps.

Step	Poor	Bad	Regular	Good	Excellent	Not realized
Insertion of trocars	1	2	3	4	5	
Flap detachment	1	2	3	4	5	
Muscle fixation	1	2	3	4	5	
Isthmotomy	1	2	3	4	5	
Thyroidectomy	1	2	3	4	5	
Location of the parathyroid glands	1	2	3	4	5	
Location of recurrent nerves	1	2	3	4	5	
Removing the part	1	2	3	4	5	
Midline closure with stitches	1	2	3	4	5	
Removing the trocars	1	2	3	4	5	

Source: Elaborated the authors.

### Statistical analysis

The data was expressed as absolute and percentage frequencies, and the means and standard deviation of the perception of use scores were calculated. In addition, the questionnaire items were assessed for their internal consistency by calculating Cronbach’s alpha. All the analyses were conducted using Statistical Package for the Social Sciences v20.0 for Windows with a 95% confidence level.

## Results

### Simulator development

**Figure 3:**
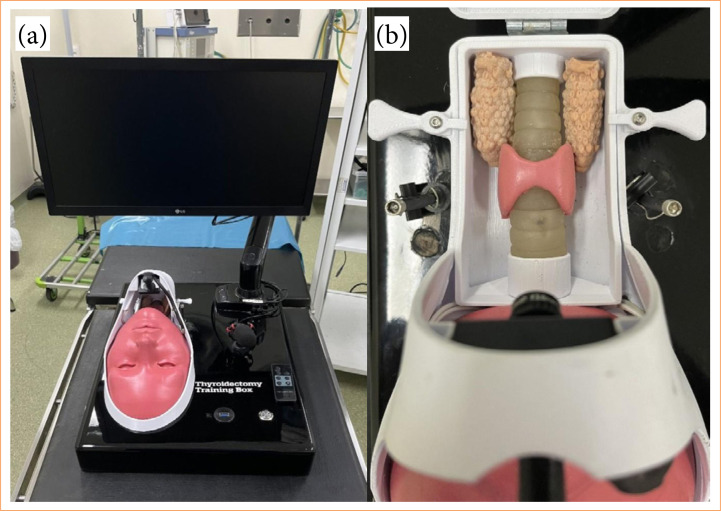
Simulator in its final phase. **(a)** External part with the mannequin’s bust and the video *set*; **(b)** internal part with some structures similar to internal organs.

Source: Elaborated the authors.

**Figure 4:**
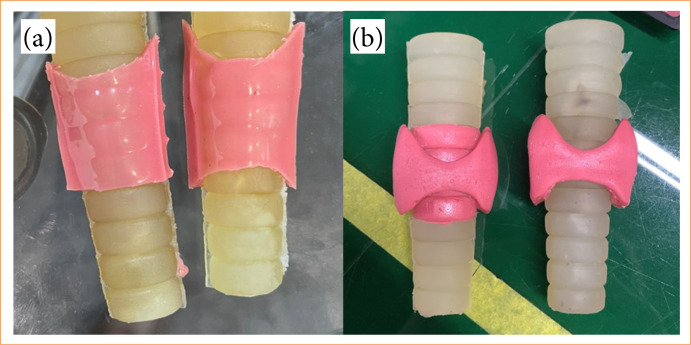
Thyroid models. **(a)** Primary thyroid model presented at the Rio de Janeiro congress; **(b)** second (thicker) model presented for the experiment at the João Pessoa Congress.

Source: Elaborated the authors..

**Figure 5:**
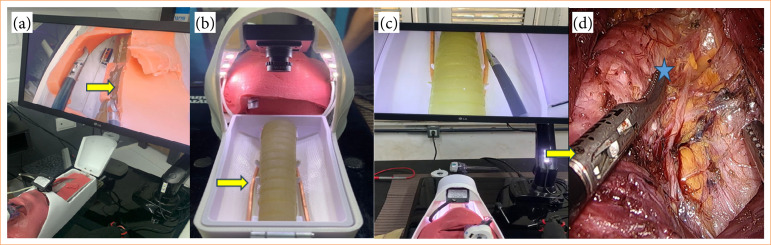
Modeling the recurrent laryngeal nerve. **(a)** Primary model of the nerve (arrow) with nylon thread; **(b)** second model made from copper wire (arrows); **(c)** demonstration of identification of the nerve using a light signal (arrow); **(d)** demonstration of the nerve (arrows) dissected next to the trachea (star) during a trans oral endoscopic thyroidectomy vestibular approach on a live patient.

Source: Elaborated the authors.

It took eight versions and four tridimensional prints of the models to reach the final version, from the development of the prototype to the minimum viable product (MVP). The simulator presented at the time of the final assessment consisted of:

- Mobile (console) for the mannequin’s support base, made of fiberglass;
- 22” LCD monitor, Samsung brand, with docking device at the back of the console, reclining for easy transportation and storage;
- Minicamera with 3.6 mm lens, zoom, light source, with view illuminated by light-emitting diode (LED) strips, attached to the rod located on the topography of the chin;
- Mannequin (cephalic part, Fig. 3b) with a larger format made of TPE for better handling and insertion of the clamps and trocars. The rod for the camera has been elevated for better visualization of the work area;
- Mannequin (cervical part, Fig. 3b) with a larger format made on a tridimensional printer with a movable roof for two-dimensional visualization of the internal structures, also making it possible to remove and place materials;
- Structures (internal part) containing a first layer plate covered with TPE to simulate the pre-thyroid musculature with a more adjusted and fixed mold, a thyroid with more thickness (Fig. 4), rods to simulate the parathyroid glands and a trachea;
- Copper wires attached to mimic the recurrent laryngeal nerve;
- Electronic components, such as an LED lamp coupled to contact sensors with copper wires (previously made of nylon wire with contact sensors) that emit a light signal when the circuit is closed to identify the recurrent nerve (Fig. 5), internally embedded electrical wiring control plug, image cable, power current stabilizer, light, camera and television switches, screws, solder, glue, etc.

### Sample characteristics

The sample that carried out the experiment with the TOETVA simulator was initially made up of 11 surgeons; however, one was excluded from the study because s/he did not meet the minimum requirement of 10 TOETVAs. The participants were mostly male, right-handed surgeons who had already completed their residency in head and neck surgery and ranged in age from 32 to 53 years old, with a mean of 42 ± 7. Around 60 percent of the participants performed manual activities, played an instrument, or played video games (Table 3).

**Table 3:** Profile of the participants.

Profile	Mean ± standard deviation	N (%)
**Sex**		
Female		1 (10)
Male		9 (90)
**Age**	42 ± 7	
**Graduation time**	18 ± 8	
**Time spent training in general surgery**	15 ± 8	
**Head-neck time**	12.5 ± 8	
**Dominant hand**		
Right		9 (90)
Both		1 (10)
**Plays a musical instrument**		
No		4 (40)
Yes		6 (60)
**Video games**		
No		4 (40)
Yes		6 (60)
**Video game time spent (hour)**		
1–2		8 (80)
4 or more		2 (20)
**Manual hobbies**		
No		9 (90)
Yes		1 (10)

Source: Elaborated the authors.

Concerning previous experience with video surgery since general surgery, only one participant had no contact with endoscopic surgery before his head and neck residency. However, during subspecialty training, around 70% had experienced some type of video surgery. The most common surgeries performed up to the questionnaire was administered were transoral thyroidectomy by video or robotic, pharyngectomy, and neck dissection (Table 4).

**Table 4:** Profile of participants in relation to experience with training and video surgery.

Profile	Mean ± standard devition	n (%)
Video surgeries before HNS residency (n/week)	13.40 ± 14.69	
Human cadaver surgery training (hours)	9.40 ± 11.20	
Animal model surgery training (hours)	13.40 ± 30.62	
Non-live simulator surgery training (hours)	36.80 ± 45.28	
Video surgeries during HNS residency		
Yes		7 (70)
No		3 (30)
MIVAT		2 (20)
Cervical drainage		1 (10)
Parotidectomy		1 (10)
Submandibulectomy		1 (10)
Thyroglossalcyst		1 (10)
Oropharyngealsurgery		1 (10)
Parathyroidectomy		1 (10)
TOETVA		1 (10)
Other		8 (80)
Video surgeries after HNS residency		10 (100)
TOETVA		10 (100)
Cervical drainage		7 (70)
Parathyroidectomy		7 (70)
Oropharyngealsurgery		8 (80)
MIVAT		1 (10)
Submandibulectomy		4 (40)
TORS		6 (60)
TORT		7 (70)
Thyroglossalductcyst		2 (20)
Other		3 (30)

Data expressed as mean ± standard deviation or absolute and percentage frequency; HNS: head and neck surgery; MIVAT: minimally invasive video-assisted thyroidectomy; TOETVA: trans oral endoscopic thyroidectomy vestibular approach; TORS: transoral robotic surgery; TORT: transoral robotic thyroidectomy. Source: Elaborated the authors.

Regarding the type of transoral thyroid surgery, 60% have performed more than 50 procedures, 30% between 20 and 50, and only 10% have performed up to 20 surgeries. Half the surgeons maintain a good percentage (20%) of video surgeries in their daily clinical practice, with a weekly average of at least one TOETVA. In contrast, open (conventional) thyroidectomy still has a lot of space among specialists, with a weekly average of four or five cases (Table 5).

**Table 5:** Profile of participants in terms of the types of simulators they have tried and their current situation regarding video surgeries.

Profile	n (%)
Cadaversurgery training	7 (70)
Animal modelsurgery training	7 (70)
Surgery training on a non-living simulator	10 (100)
Training modelpreferred	
Animal model	3 (30)
Humancorpse	6 (60)
Virtual simulator	1 (10)
Head and neck video surgery course	10 (100)
TOETVAs already held	
10–20	1 (10)
20–50	3 (30)
50+	6 (60)
Percentage of video procedures in HNS	26.50 ± 26.88
Robotic head and neck procedures week	0.70 ± 0.82
RoboticHead and neck procedures month	3.20 ± 2.25
Video thyroidectomies per week	0.60 ± 0.70
Videothyroidectomies per month	2.80 ± 1.75
Conventionalthyroidectomies per week	4.60 ± 2.99
Conventionalthyroidectomies per month	18.10 ± 12.93
Would you prefer the simulator with the camera on the trocar and an assistant surgeon?	
No	3 (30)
Yes	7 (70)

Data expressed as mean ± standard deviation or absolute and percentage frequency; TOETVA: trans oral endoscopic thyroidectomy vestibular approach; HNS: head and neck surgery. Source: Elaborated the authors.

Concerning experience in courses and simulators, all the surgeons had already undergone at least one training course, which facilitated adaptation and enhanced the evaluation of the experiment and the simulator. Out of the participants, 70% had already experienced endoscopic/robotic surgery training with a human cadaver, 60% with an animal model, and 100% with non-living models. In this case, training on human cadavers was the most preferred type (60%), because it was more similar to reality (Table 5).

Despite this great benefit of better simulating real surgery, the type of simulation that generated the most hours of training for the participants was realistic simulation with non-living simulators, averaging 36 hours per participant, in contrast to the average of 9 hours with a human cadaver.

When asked if they would prefer an assistant guiding the camera to fully simulate the surgery, 60% said they would like this option.

### Simulator evaluation: final version

No one rated it as very bad or bad on the Likert scale. Everyone rated the simulator as fair, good, or excellent on all the items asked.

Points such as the ability to simulate a TOETVA, synthetic structures, surgical material, design, ergonomics, visibility of the operating field, resistance, resilience, *fulcrum* effect, and practicality obtained 100% of responses between good and excellent. The best evaluations, with the most votes for excellent, were for practicality, ergonomics, and surgical material (Table 6).

**Table 6:** Overall evaluation of the simulator.

Simulator overview – TTB	Mean ± SD	Cronbach’s alpha	Likertscale			
			Bad	Regular	Good	Excellent
Simulate a TOETVA	4.50 ± 0.53	0,833	0 (0)	0 (0)	5 (50)	5 (50)
Synthetic structures	4.40 ± 0.52	0,827	0 (0)	0 (0)	6 (60)	4 (40)
Tweezers and surgical equipment	4.70 ± 0.48	0,820	0 (0)	0 (0)	3 (30)	7 (70)
Videoequipment	4.20 ± 0.79	0,833	0 (0)	2 (20)	4 (40)	4 (40)
Visual appearance (looks like real)	4.50 ± 0.53	0,802	0 (0)	0 (0)	5 (50)	5 (50)
Simulator design	4.60 ± 0.52	0,804	0 (0)	0 (0)	4 (40)	6 (60)
Adequate depth	4.60 ± 0.70	0,847	0 (0)	1 (10)	2 (20)	7 (70)
Ergonomics and positioning	4.80 ± 0.42	0,820	0 (0)	0 (0)	2 (20)	8 (80)
Visibility of the operating field	4.30 ± 0.48	0,845	0 (0)	0 (0)	7 (70)	3 (30)
Material strength	4.40 ± 0.52	0,802	0 (0)	0 (0)	6 (60)	4 (40)
Material resilience	4.60 ± 0.52	0,820	0 (0)	0 (0)	4 (40)	6 (60)
Fulcrumeffect	4.80 ± 0.42	0,818	0 (0)	0 (0)	2 (20)	8 (80)
Practicality	4.90 ± 0.32	0,818	0 (0)	0 (0)	1 (10)	9 (90)

Data expressed as mean ± standard deviation (SD) or absolute and percentage frequency; TOETVA: trans oral endoscopic thyroidectomy vestibular approach; TTB: thyroidectomy training box. Source: Eaborated the authors.

The characteristics that received at least one regular vote were video equipment, visual appearance (looking like the real thing), and depth adequacy (Table 6).

Finally, the means and standard deviation of the perception of use scores were calculated and are shown in Table 6.

Cronbach’s alpha is a coefficient that measures the internal consistency of a questionnaire, and the desired value is usually between 0.8 and 0.9 to be considered high reliability15. Thus, the alpha value found was greater than 0.8 for all the answers.

### Evaluation of the surgical stages

The surgery *checklist* and the simulator manual are products developed to help memorize and standardize the TOETVA step-by-step procedure. After performing several transoral thyroidectomies, together with the exchange of experience with the biggest names in this surgery in Brazil, we were able to arrive at what is shown in Table 2.

To complete the idealization of the model, some stages of the surgery still need to be simulated, such as detaching the flap, making the *pocket*, and the *endobag*. Other steps on the checklist may not be adapted to the simulator, as they are not unanimous among the most experienced surgeons or because they are too costly to adopt, such as closing the midline and creating the *pocket*, respectively.

Thus, the steps that were not carried out in the experiment were:

- Making the *pocket*;
- Flap detachment;
- Ligation of the upper pedicle;
- Recurrent emptying;
- Making the *endobag*;
- Closing the midline with dots.

With regard to the stages evaluated, three stood out with more evaluations between good and excellent: isthmotomy, thyroidectomy, and midline opening (Table 7).

**Table 7:** Overall evaluation of the simulation.

Step-by-step surgery on the simulator	Mean ± SD	Cronbach’s α	Likertscale			
			Bad	Regular	Good	Excellent
Insertion of trocars	3.60 ± 0.97	0.842	1 (10)	4 (40)	3 (30)	2 (20)
Midline opening	4.60 ± 0.70	0.802	0 (0)	1 (10)	2 (20)	7 (70)
Fixing the muscles with a stitch	4.57 ± 0.53	0.804	0 (0)	0 (0)	3 (42.9)	4 (57.1)
Isthmotomy	4.90 ± 0.32	0.818	0 (0)	0 (0)	1 (10)	9 (90)
Thyroidectomy	4.60 ± 0.52	0.802	0 (0)	0 (0)	4 (40)	6 (60)
Location of the parathyroid glands	4.40 ± 0.70	0.802	0 (0)	1 (10)	4 (40)	5 (50)
Location of the recurrent laryngeal nerve	4.00 ± 0.94	0.803	1 (10)	1 (10)	5 (50)	3 (30)
Making the *Endobag*	4.00 ± 1.41	0.814	0 (0)	1 (25)	1 (25)	2 (50)
Part removal with *Endobag*	4.14 ± 1.21	0.803	1 (14.3)	1 (14.3)	1 (14.3)	4 (57.1)
**Adjusted average (0–100)**	**85.72 ± 5.43**	**0.825**				

Data expressed as mean ± standard deviation (SD) or absolute and percentage frequency. Source: Elaborated the authors.

The insertion of the trocars and the location of the recurrent laryngeal nerve received one (10%) bad vote, but 60 and 80% were rated as good or excellent, respectively (Table 7).

All the steps tested had a Cronbach’s alpha value greater than 0.8, with a final average of 85% acceptance.

## Discussion

This study was based on the need to create a realistic and efficient non-living simulator as a viable and affordable alternative for training surgeons in an alternative technique for thyroid removal, TOETVA. The sample of surgeons used to validate the simulator showed people who were extremely skilled in the technique, with 90% of the participants having carried out more than 20 procedures, which makes the validation test robust, as they well exceed the numbers suggested for a good learning curve^8^.

As there is no non-live simulator of transoral thyroid surgery in the world literature, at least until the date of publication of this study, the simulator is an important tool for developing skills in this technique for new surgeons or for more experienced surgeons who have no training in videoendoscopic surgery. Given this, there is no way of comparing it with other TOETVA simulators yet. Comparisons should be made with other available simulators, especially in the validation stages tested. In this sense, most published thyroidectomy simulations are performed on a human or animal cadaver^16^. Therefore, the surgical steps followed in thyroidectomy training box were similar to those of other cadaver models of this surgery^17^.

It can be also seen that, concerning visual and content validations, the simulator in this study received excellent evaluations with scores always above 4.0 on average on the Likert scale, which is in line with higher evaluations than other validated and tested simulators from other areas^18^^–^ 20.

The simulator was tested in the main fields of competence that a simulator must pass to acquire efficient validation: visual validity (*face validity*), and content validity (*content validity*)^18^.

Regarding visual appearance and design, at least 90% of respondents rated it as good or excellent. All even rated the ability to simulate a TOETVA as good or excellent. Ergonomics, depth, and visibility of the operating field also received a high level of acceptance. Thus, visual validity, which refers to the extent to which the simulator resembles a realistic environment, received excellent evaluations. Regarding content validity, which describes the simulator’s ability to serve as a surgical model, 90% of the experts voted the simulator’s practicality as excellent, in addition to excellent acceptance of the synthetic structures, video, and surgical material used.

Among the disadvantages of a cadaver surgery model are the high cost and the number of repetitions that can be carried out, as well as the need for a complete and expensive structure to support the necessary ethical and legal care^21^. In view of the above, thyroidectomy training box has advantages in that it reduces costs and increases the possibility of performing more surgeries in a single model, as well as there being no ethical questions about it.

The simulator closely resembles a patient’s cervix, with appropriate adaptation of cervical hyperextension and ergonomics to simulate the reality of surgery. It is equipped with a fixed high-definition video camera to facilitate training when the surgeon does not have an assistant. In addition, the simulator developed also has the option of recording and broadcasting the procedure live, as well as a microphone for voice capture, which makes it possible to correct and adjust the surgery both afterward and in real time between the preceptor and the student. There is also the possibility of coupling robotic platforms, making transoral robotic thyroidectomy training possible.

In this study, despite the extensive experience of the participants, it was possible to generate reflection and applicability in what was practiced through exchange and feedback between the participants and the author, which, according to the stages above, is part of the learning process. Some surgeons, for example, were able to practice endoscopic suturing, a step that is not essential for performing surgery, but may be necessary in the event of an intercurrence or adverse situation.

The model used (checklist and video) could be applied in training models to ensure a learning curve of less than 10–15 TOETVAs^22^^,^ 23, especially considering that head and neck surgeons with no experience in robotic or laparoscopic surgery have a longer learning curve, up to 30 surgeries^17^. Thus, a TOETVA preparation structure is suggested to implement safe training that includes sufficient observation, cadaver dissection, video observation, and supervision by a mentor^24^.

To be aware, in Brazil, around 65% of the surgeons who already perform TOETVA are in the Southeast^12^, making the learning curve difficult with the presence of a more experienced surgeon, both due to the concentration of tutors in one region and the distance between Brazilian states. In this way, the simulator can serve as a training alternative for those who do not have the financial or travel facilities to attend courses or get help from preceptors.

Among the limitations of the study, there are:

- The small sample size, but with good relative representativeness, as there are not many trained surgeons with extensive experience throughout Brazil. The projection is that there will be just over 120 people performing this surgery in Brazil by the beginning of 2023^12^;
- It was not possible to reproduce all the steps of the surgery for reasons of logistics and lack of funding, as the insertion of any step generates a lot of development and manufacturing costs;
- Neither construct nor translation validations were carried out. The first would be conducted by creating comparative groups between the results of more experienced surgeons and those of apprentices. The second would consist of evaluating the ability to obtain improvements in surgery in human beings before and after training with the simulator.

By addressing these limitations, new opportunities will arise to improve the simulator, providing additional benefits to the individuals who will use it for training. This evolution could also positively influence the integration of the simulator into the head and neck surgery residency curriculum, representing a significant advance in the field of surgical education.

## Conclusion

The simulator developed could be used to train new surgeons in endoscopic thyroid surgery, as well as experienced surgeons. The thyroidectomy training box was carefully evaluated by surgeons, resulting in a robust validation test. These professionals tested the main fields of competence, which are visual validity (*face validity*) and content validity (*contentvalidity*).

In this way, the simulator, with the formulation of a standardized curriculum (*checklist* and surgical videos), can help with the learning and standardization of TOETVA.

## Data Availability

All data sets were generated or analyzed in the current study.

## References

[1] Hannan SA (2006). The magnificent seven: a history of modern thyroid surgery. Int J Surg.

[2] Goswami S, Peipert BJ, Mongelli MN, Kurumety SK, Helenowski IB, Yount SE, Sturgeon C (2019). Clinical factors associated with worse quality-of-life scores in United States thyroid cancer survivors. Surgery.

[3] Juarez MC, Ishii L, Nellis JC, Bater K, Huynh PP, Fung N, Darrach H, Russell JO, Ishii M (2019). Objectively measuring social attention of thyroid neck scars and transoral surgery using eye tracking. Laryngoscope.

[4] Miccoli P, Berti P, Conte M, Bendinelli C, Marcocci C (1999). Minimally invasive surgery for thyroid small nodules: preliminary report. J Endocrinol Invest.

[5] Miccoli P, Fregoli L, Rossi L, Papini P, Ambrosini CE, Bakkar S, De Napoli L, Aghababyan A, Matteucci V, Materazzi G (2020). Minimally invasive video-assisted thyroidectomy (MIVAT). Gland Surg.

[6] Witzel K, von Rahden BH, Kaminski C, Stein HJ (2008). Transoral access for endoscopic thyroid resection. Surg Endosc.

[7] Benhidjeb T, Wilhelm T, Harlaar J, Kleinrensink GJ, Schneider TA, Stark M (2009). Natural orifice surgery on thyroid gland: totally transoral video-assisted thyroidectomy (TOVAT): report of first experimental results of a new surgical method. Surg Endosc.

[8] Anuwong A, Ketwong K, Jitpratoom P, Sasanakietkul T, Duh QY (2018). Safety and outcomes of the transoral endoscopic thyroidectomy vestibular approach. JAMA Surg.

[9] Anuwong A (2016). Transoral endoscopic thyroidectomy vestibular approach: a series of the first 60 human cases. World J Surg.

[10] Bertelli AAT, Rangel LG, Lira RB, Tesseroli MAS, Santos IC, Silva GD, Gomes MA, Tenório LR, Kowalski LP, Gonçalves AJ, Russel JO, Tufano RP (2021). Trans oral endoscopic thyroidectomy vestibular approach (TOETVA) in Brazil: safety and complications during learning curve. Arch Endocrinol Metab.

[11] Vallas C, Alexiou K, Alexandrou A, Economou N (2014). Different forms of laparoscopic training: review and comparison. Hellenic J Surg.

[12] Tenório LR, Bertelli AA, Nakai MY, Menezes MB, Russell JO, Gonçalves AJ (2023). Transoral thyroid and parathyroid surgery in Brazil: where are we?. Rev Col Bras Cir.

[13] Cooke RM, Probst KN (2006). Highlights of the Expert Judgment Policy Symposium and Technical Workshop.

[14] Knol AB, Slottje P, van der Sluijs JP, Lebret E (2010). The use of expert elicitation in environmental health impact assessment: a seven step procedure. Environ Health.

[15] Streiner DL (2003). Being inconsistent about consistency: when coefficient alpha does and doesn’t matter. J Pers Assess.

[16] Razavi CR, Tanavde V, Shaear M, Richmon JD, Russell JO (2020). Simulations and simulators in head and neck endocrine surgery. Ann Thyroid.

[17] Richmon JD, Pattani KM, Benhidjeb T, Tufano RP (2011). Transoral robotic-assisted thyroidectomy: a preclinical feasibility study in 2 cadavers. Head Neck.

[18] Varoquier M, Hoffmann CP, Perrenot C, Tran N, Parietti-Winkler C (2017). Construct, face, and content validation on Voxel-Man® simulator for otologic surgical training. Int J Otolaryngol.

[19] Ramos P, Montez J, Tripp A, Ng CK, Gill IS, Hung AJ (2014). Face, content, construct and concurrent validity of dry laboratory exercises for robotic training using a global assessment tool. BJU Int.

[20] Leijte E, Arts E, Witteman B, Jakimowicz J, De Blaauw I, Botden S (2019). Construct, content and face validity of the eoSim laparoscopic simulator on advanced suturing tasks. Surg Endosc.

[21] Khafif A, Cohen O, Masalha M, Yaish I, Hod K, Assadi N (2021). Adoption of the transoral endoscopic vestibular approach by head and neck surgeons without prior laparoscopic/robotic experience. Head Neck.

[22] Lira RB, Ramos AT, Nogueira RMR, de Carvalho GB, Russell JO, Tufano RP, Kowalski LP (2020). Transoral thyroidectomy (TOETVA): Complications, surgical time and learning curve. Oral Oncol.

[23] BERTELLI Antonio Augusto Tupinamba (2019). Transoral endoscopic thyroidectomy by vestibular approach (TOETVA): initial experience in anacademic hospital. Arch Head NeckSurg.

[24] Lee DW, Bang HS, Jeong JH, Kwak SG, Choi YY, Tae K (2021). Cosmetic outcomes after transoral robotic thyroidectomy: Comparison with transaxillary, postauricular, and conventional approaches. Oral Oncol.

